# Research on Early-Age Shrinkage and Prediction Model of Ultra-High-Performance Concrete Based on the BO-XGBoost Algorithm

**DOI:** 10.3390/ma19081624

**Published:** 2026-04-17

**Authors:** Fang Luo, Jun Wang, Chenhui Zhu, Jie Yang

**Affiliations:** 1 School of Civil Engineering, Zhengzhou College of Finance and Economics, Zhengzhou 450044, China; 2 Management Division of Tongyu River Qiangwei River and Clear Water Delivery Conservancy Project, Huaian 223001, China; 3 School of Transportation and Civil Engineering, Nantong University, Nantong 226019, China

**Keywords:** ultra-high-performance concrete, early-age shrinkage, XGBoost, Bayesian optimization, SHAP

## Abstract

Early-age shrinkage is a critical factor governing the dimensional stability and cracking susceptibility of ultra-high-performance concrete (UHPC). However, accurate prediction of UHPC shrinkage remains challenging due to the strong nonlinear interactions among mixture parameters, curing conditions, and hydration-induced internal moisture evolution, particularly when only limited experimental data are available. In this study, a systematic experimental program was conducted to investigate the influence of the binder-to-sand ratio, water-to-binder ratio, polypropylene fiber dosage, and curing environment on both early drying shrinkage and autogenous shrinkage of UHPC. Based on the experimental results, a structured dataset covering all shrinkage test data was constructed to support data-driven modeling. To improve prediction reliability under small-sample conditions, a Bayesian-optimized Extreme Gradient Boosting (BO-XGBoost) framework was developed and benchmarked against several conventional machine learning models, including Backpropagation Neural Networks (BPNNs), Random Forest (RF), and Support Vector Machines (SVMs). Shrinkage test data from other literature validated the prediction accuracy of this model, demonstrating its rationality and practicality. In addition, the Shapley Additive Explanations (SHAP) method was employed to quantitatively interpret the contribution and interaction mechanisms of key variables affecting shrinkage behavior. The results show that the BO-XGBoost model achieves the highest prediction accuracy and stability among the evaluated algorithms. SHAP analysis further reveals that curing age and curing environment dominate drying shrinkage, whereas autogenous shrinkage is primarily governed by the curing age and water-to-binder ratio. The interaction analysis also identifies the coupled effects between low water-to-binder ratio and extended curing age. The proposed framework not only improves prediction robustness for UHPC shrinkage under limited data conditions but also provides interpretable insights into the mechanisms governing early-age deformation. These findings offer a data-driven basis for optimizing UHPC mixture design and mitigating early-age cracking risks in engineering applications.

## 1. Introduction

Ultra-high-performance concrete (UHPC), developed on the basis of dense particle packing theory [1,2], has attracted increasing attention in recent years due to its exceptional mechanical strength, compact microstructure, and superior durability. As a representative advanced cement-based material, UHPC has been widely applied in bridge engineering, prefabricated structural elements, and marine infrastructure [3,4]. However, the typical composition of UHPC, characterized by a very low water-to-binder ratio and a high binder content, inevitably intensifies early-age shrinkage during hydration and hardening. Excessive early-age shrinkage may lead to the accumulation of internal tensile stresses and the initiation of microcracks, thereby negatively affecting structural reliability, dimensional stability, and long-term durability [5]. Previous studies have shown that in UHPC systems with very low water-to-binder ratios, early-age shrinkage can account for a significant proportion of total shrinkage. Therefore, understanding and accurately predicting the early-age shrinkage behavior of UHPC is essential for optimizing mixture design and mitigating early-age cracking risks.

Current research on UHPC early-age shrinkage has mainly focused on the effects of mixture parameters such as water-to-binder ratio, mineral admixtures, and fiber content, with particular emphasis on autogenous shrinkage mechanisms [6,7,8]. Nevertheless, early-age shrinkage of UHPC is governed not only by mixture composition but also by complex interactions among hydration kinetics, pore structure evolution, internal humidity reduction, and external curing conditions [9]. These coupled physicochemical processes introduce strong nonlinearity and uncertainty into shrinkage development. Under such circumstances, traditional empirical models or semi-analytical approaches often struggle to capture the multivariate coupling effects among key variables, resulting in limited prediction accuracy and restricted applicability when mixture parameters or curing conditions vary.

In recent years, machine learning techniques have demonstrated strong potential in modeling complex behaviors of cement-based materials. Ensemble learning algorithms, such as Extreme Gradient Boosting (XGBoost), have shown excellent capability in capturing nonlinear relationships in multiparameter material systems. Previous studies have successfully applied machine learning approaches to predict compressive strength, durability indicators, and structural performance of concrete and UHPC [10,11,12,13,14,15]. Furthermore, interpretable machine learning methods combined with SHAP analysis have been increasingly adopted to quantify the influence of individual variables and their interactions in cementitious materials systems [16,17,18]. Hybrid machine learning frameworks integrating optimization algorithms have also been proposed to enhance prediction accuracy and model robustness in concrete property prediction [19,20].

Despite these advances, the application of machine learning methods to the prediction of early-age shrinkage behavior in UHPC remains relatively limited. Compared with properties such as compressive strength, systematic experimental datasets for UHPC shrinkage are still scarce, and the complex coupling between mixture design parameters and environmental conditions further complicates shrinkage prediction. In addition, many existing machine learning studies rely on relatively large datasets, whereas experimental studies on UHPC often involve limited sample sizes due to the high cost and complexity of testing. Under such small-sample conditions, conventional machine learning models may suffer from reduced prediction stability and generalization capability.

Bayesian optimization provides an efficient strategy for improving the performance of machine learning models by automatically searching for optimal hyperparameter combinations [21,22,23]. By constructing a probabilistic surrogate model of the objective function and iteratively balancing exploration and exploitation, Bayesian optimization can efficiently identify near-optimal parameter configurations with a limited number of evaluations [24,25,26,27]. When integrated with gradient boosting algorithms such as XGBoost, Bayesian optimization has been shown to improve prediction robustness and model generalization in complex engineering datasets [28,29].

Therefore, this study aims to develop an interpretable data-driven framework for predicting early-age shrinkage behavior of UHPC under limited experimental data conditions. A systematic experimental database was first established by investigating the effects of binder-to-sand ratio, water-to-binder ratio, polypropylene fiber dosage, curing environment, and curing age on both drying shrinkage and autogenous shrinkage of UHPC. Based on this dataset, a Bayesian-optimized Extreme Gradient Boosting (BO-XGBoost) shrinkage deformation prediction model was developed to enhance prediction accuracy and robustness under small-sample conditions. Furthermore, the SHAP method was introduced to quantitatively interpret the relative importance and interaction mechanisms of the governing variables, enabling a direct connection between machine learning predictions and the physical mechanisms controlling shrinkage behavior.

## 2. Materials and Methods

### 2.1. Materials and Mix Proportion

**Figure 1:**
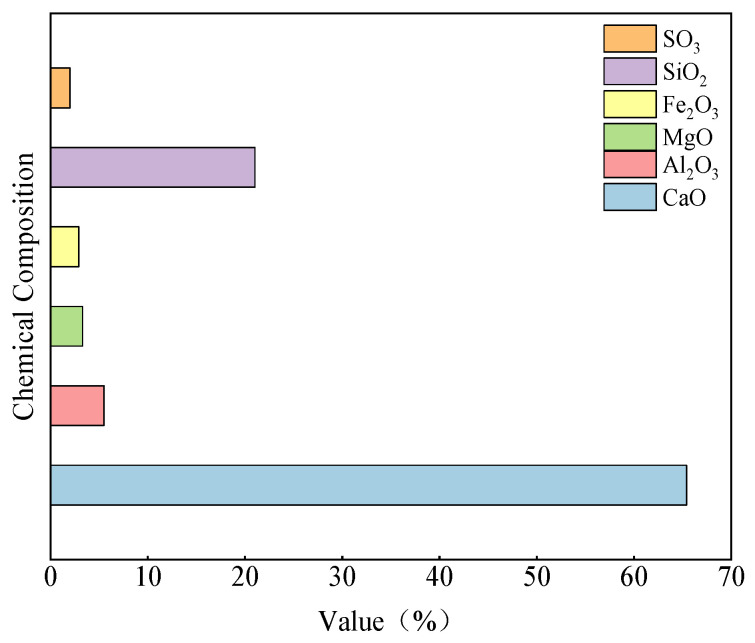
The chemical composition.

The fine aggregate used in this study was natural river sand with a bulk density of 1560 kg/m^3^ and a fineness modulus of 2.68. Ordinary Portland cement (PC 42.5) served as the primary cementitious material, and its chemical composition is presented in Figure 1. Grade I silica fume was incorporated as a mineral admixture. Polypropylene (PP) fiber was used as the non-metallic fiber component; it was a short monofilament plastic fiber with a length of 6 mm, a diameter of 30 μm, and a tensile strength of 780 MPa. In addition, straight copper-coated steel fibers with a length of 13 mm, a diameter of 350 μm, and a tensile strength of 2.8 GPa were incorporated to enhance the mechanical performance of UHPC. A polycarboxylate-based superplasticizer in powder form was used as the chemical admixture, providing a water-reduction efficiency exceeding 25%.

To systematically investigate the influence of mixture parameters on the early-age shrinkage behavior of UHPC, it is necessary to clarify the basis for selecting the key variables. The binder-to-sand ratio and water-to-binder ratio directly determine the paste volume and pore structure characteristics, which are fundamental factors governing both autogenous shrinkage and drying shrinkage. Polypropylene fiber content was included because fibers play a key role in restraining early-age deformation and microcrack development. Curing environment and curing age reflect the external conditions and the time-dependent evolution of hydration and moisture transport, both of which jointly govern the kinetics of shrinkage development. In addition, water content and sand content were incorporated as they represent the absolute amounts of key constituents that directly affect the internal humidity and internal restraint capacity of the composite system. Other factors, such as the type and dosage of supplementary cementitious materials (e.g., silica fume, fly ash), chemical admixtures, and temperature conditions, can also influence the shrinkage behavior of UHPC. However, the primary objective of this study is to establish a systematic framework for predicting early-age shrinkage based on a carefully controlled experimental design, focusing on the most commonly adjusted parameters in UHPC mix design. Through a systematic investigation of the above seven variables, this study aims to provide a practical and interpretable data-driven tool that can be readily adopted in engineering applications. The influence of additional factors will be gradually incorporated in future work as the experimental database expands.

Based on the above variables, ten representative UHPC mixture proportions were designed. For each mixture, eight specimens were prepared, including four specimens for drying shrinkage measurements and four specimens for autogenous shrinkage tests. The detailed mixture proportions and corresponding groupings are summarized in Table 1.

### 2.2. Experimental Design and Testing Methods

(1) Specimen preparation

**Figure 2:**
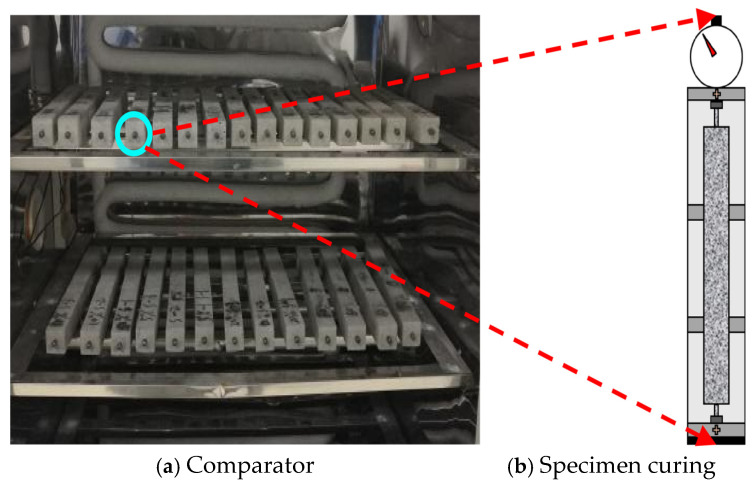
Actual image of drying shrinkage test.

Early-age drying shrinkage and autogenous shrinkage tests were conducted in accordance with the Test Method for Drying Shrinkage of Cement Mortar [30,31]. Prismatic specimens with dimensions of 25 mm × 25 mm × 280 mm were prepared, as illustrated in Figure 2. For each mixture, eight specimens were fabricated, including four specimens for drying shrinkage tests and four specimens for autogenous shrinkage measurements in order to ensure experimental reliability.

Cast-iron molds equipped with copper gauge studs were used to ensure compatibility with the length comparator requirements. During specimen preparation, the fresh mortar was placed into the molds in two layers. Each layer was compacted manually using a tamping rod and subsequently consolidated on a vibration table to minimize entrapped air. After surface finishing, all specimens were transferred to a standard curing chamber maintained at a temperature of 25 ± 2 °C and a relative humidity of 98 ± 2% for 24 h. Following demolding, the initial length of each specimen was measured immediately.

For autogenous shrinkage measurements, the demolded specimens were tightly sealed with industrial plastic film to prevent moisture exchange and then returned to the curing chamber for continuous curing up to 28 days. In contrast, specimens designated for drying shrinkage tests were exposed to the specified drying conditions after the initial curing period. Length changes were recorded at predetermined ages under identical environmental conditions.

(2) Curing and measurement

To examine the influence of environmental exposure on early-age shrinkage behavior, a dry curing condition was introduced as a reference environment. Specimens in this group were stored under ambient laboratory conditions, with the temperature maintained at (28 ± 5) °C and the relative humidity controlled at (60 ± 15)%.

**Figure 3:**
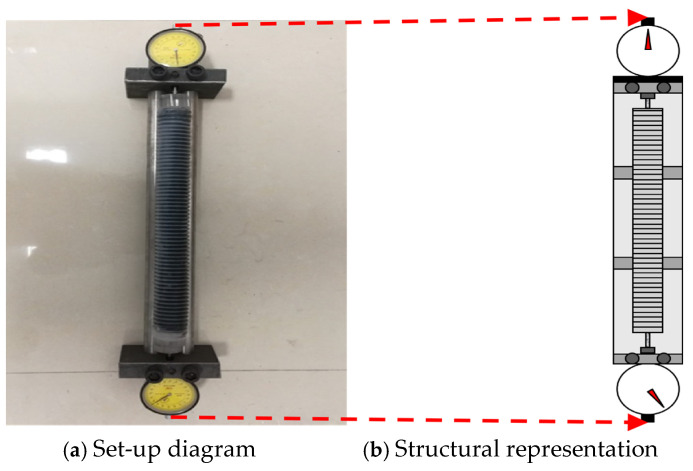
Physical appearance of the autogenous shrinkage testing device.

Length variations were monitored using a linear length comparator at short-term intervals of 0, 1, 2, 4, 6, 8, 10, and 12 h, as well as at longer curing ages of 1, 3, 5, 7, 10, 14, 21, and 28 d. Based on the measured length changes, the corresponding early drying shrinkage and autogenous shrinkage strains were calculated. The overall experimental arrangement and measurement procedure are schematically presented in Figure 2 and Figure 3.

### 2.3. Experimental Results

#### 2.3.1. Effect of Binder-to-Sand Ratio on Early-Age Shrinkage of UHPC

**Figure 4:**
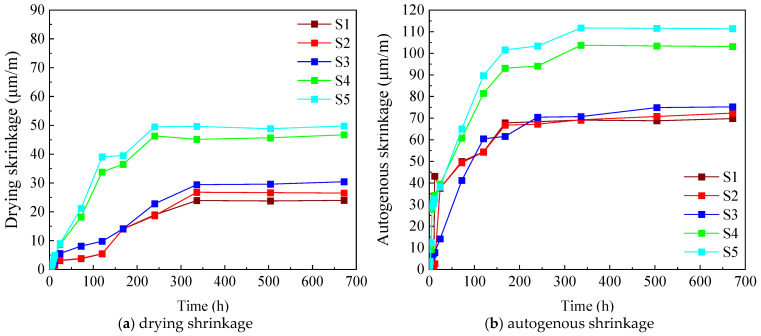
Effect of binder-to-sand ratio on early-age shrinkage of UHPC.

To clarify the effect of the binder-to-sand ratio on the early-age shrinkage behavior of UHPC, a series of mixtures was prepared with a constant water-to-binder ratio of 0.17 and binder-to-sand ratios of 0.8, 0.9, 1.0, 1.1, and 1.2. Both early drying shrinkage and autogenous shrinkage were measured, and the corresponding results are presented in Figure 4. Regardless of the binder-to-sand ratio, all UHPC mixtures exhibited a characteristic two-stage evolution of shrinkage at early ages, consisting of a rapid development stage followed by a gradually stabilizing stage. At 28 days, the drying shrinkage values increased from 23 × 10^−6^ to 52 × 10^−6^ as the binder-to-sand ratio rose from 0.8 to 1.2, while the corresponding autogenous shrinkage increased from 70 × 10^−6^ to 112 × 10^−6^. These results demonstrate a clear positive correlation between binder-to-sand ratio and both types of shrinkage, indicating that higher binder contents lead to more pronounced early-age deformation. The observed trend can be explained by two main factors. On the one hand, shrinkage of the cement paste is the primary source of volumetric deformation in UHPC. Increasing the binder-to-sand ratio raises the volume fraction of cement paste in the composite, which directly amplifies the overall shrinkage [32]. On the other hand, fine aggregates provide mechanical restraint to the surrounding paste, effectively limiting its free shrinkage. As the binder-to-sand ratio increases, the relative amount of fine aggregate decreases, resulting in reduced internal restraint and a lower composite stiffness. In addition, a higher paste content creates more continuous pore space and facilitates hydration-related transport processes, such as ion diffusion and product precipitation, which further intensify shrinkage development.

These results indicate that the early-age volume stability of UHPC is strongly dependent on the proportion of fine aggregates and that careful control of the binder-to-sand ratio is essential for mitigating drying and autogenous shrinkage.

#### 2.3.2. Effect of Water-to-Binder Ratio on Early-Age Shrinkage of UHPC

**Figure 5:**
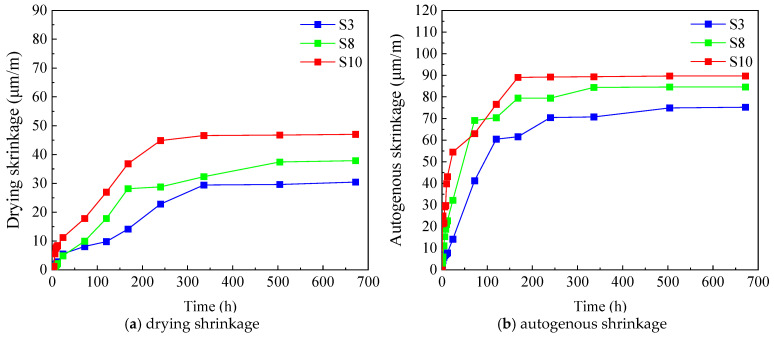
Effect of water-to-binder ratio on early-age shrinkage of UHPC.

To investigate the effect of the water-to-binder ratio (W/B) on the early-age shrinkage behavior of UHPC, drying shrinkage and autogenous shrinkage tests were carried out on specimens with W/B values of 0.15, 0.16, and 0.17, and the corresponding results are shown in Figure 5. Regardless of W/B, all specimens exhibited a typical two-stage evolution of shrinkage, characterized by an initial rapid development stage followed by a slower, decelerating stage. During the early rapid stage, intensive cement hydration combined with a sharp reduction in internal relative humidity markedly accelerated both drying and autogenous shrinkage. Under these conditions, UHPC with a lower W/B was more prone to the development of early-age microcracks [33]. Quantitative comparison further indicates that a reduction in W/B significantly increases the rate and magnitude of early shrinkage. When the W/B decreased from 0.17 to 0.15, both drying shrinkage and autogenous shrinkage increased appreciably. This behavior can be mainly attributed to changes in pore structure and moisture state induced by a lower W/B. Specifically, decreasing the W/B leads to pore structure refinement and a reduction in capillary pore size, which in turn increases capillary tension and intensifies shrinkage deformation [34]. In addition, a lower W/B limits the amount of free water available for hydration, causing a more rapid decline in internal relative humidity and an earlier onset of self-desiccation, thereby promoting autogenous shrinkage. Overall, reducing the water-to-binder ratio exacerbates early-age volumetric shrinkage of UHPC by altering capillary pore characteristics and internal moisture distribution, highlighting W/B as a key mix-design parameter governing the volumetric stability of UHPC [35].

#### 2.3.3. Effect of Fiber Type and Dosage on Early-Age Shrinkage of UHPC

**Figure 6:**
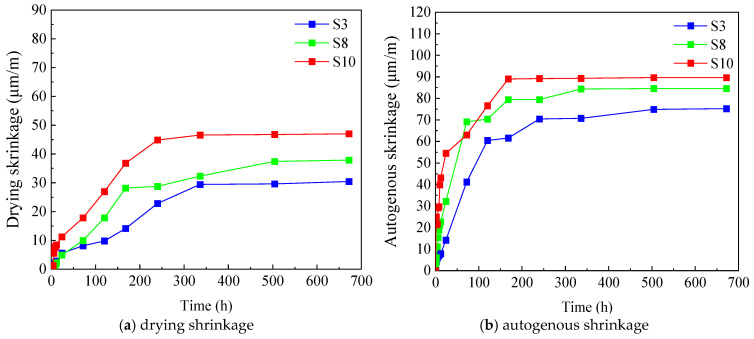
Effect of fiber types and dosage on early-age shrinkage of UHPC.

To assess the effect of hybrid steel–polypropylene (PP) fibers on the early-age shrinkage behavior of UHPC, three groups of specimens were prepared with a constant steel fiber volume fraction of 2.0% and PP fiber contents of 0.05%, 0.10%, and 0.15%. Early drying shrinkage and autogenous shrinkage were measured, and the corresponding results are presented in Figure 6. At 28 days, the drying shrinkage strain and autogenous shrinkage strain of UHPC first decreased and then increased with the increase in PP fiber content. Among them, the mixture containing 0.10% PP fiber exhibited the lowest values for both drying and autogenous shrinkage, indicating that an appropriate PP fiber dosage can effectively alleviate early-age shrinkage deformation. This beneficial effect can be explained by two main mechanisms. On the one hand, the combined action of steel and PP fibers improves particle interlocking within the matrix and enhances frictional resistance at the fiber–matrix interface, which facilitates a more uniform redistribution of shrinkage-induced stresses. On the other hand, the randomly oriented hybrid fibers form a spatial reinforcing network capable of bearing part of the internal tensile stress, thereby lowering the effective stress level in the cementitious matrix. This “bridging and passivation” effect delays microcrack initiation and restrains crack propagation during early shrinkage.

When the PP fiber content exceeded 0.10%, however, both drying shrinkage and autogenous shrinkage increased. This adverse trend is mainly attributed to fiber agglomeration at higher dosages, which compromises fiber dispersion and weakens their restraining efficiency. In addition, PP fibers exhibit a certain degree of water absorption; excessive fiber incorporation effectively reduces the available free water in the system, accelerates internal self-desiccation, and thus intensifies autogenous shrinkage [36]. Overall, the results demonstrate that a moderate amount of PP fiber, when used in combination with steel fibers, can significantly enhance the early-age volumetric stability of UHPC. In contrast, excessive PP fiber addition leads to dispersion-related defects and increased water demand, ultimately diminishing shrinkage-mitigation effectiveness. This confirms the existence of an optimal PP fiber dosage range for achieving improved shrinkage control in UHPC.

#### 2.3.4. Effect of Curing Condition on Early-Age Shrinkage of UHPC

**Figure 7:**
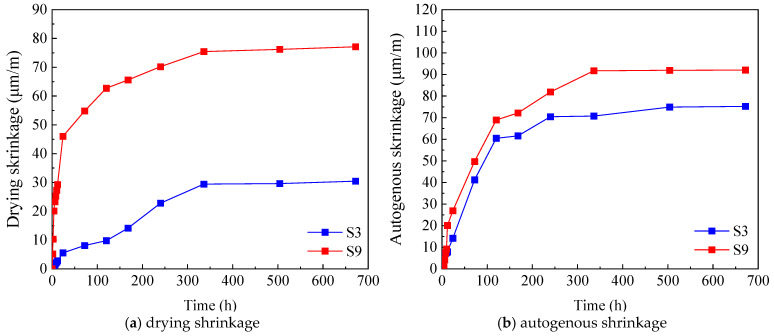
Effect of curing condition on early-age shrinkage of UHPC.

To elucidate the effect of curing conditions on the early-age shrinkage behavior of UHPC, drying shrinkage and autogenous shrinkage tests were carried out under standard curing and dry curing regimes, with the results shown in Figure 7. The comparison clearly indicates that both types of shrinkage were substantially higher under dry curing than under standard curing. The drying shrinkage strain of UHPC under dry curing was significantly larger than that under standard curing, and a comparable trend was observed for autogenous shrinkage, which was also notably higher under dry curing. The pronounced difference between the two curing regimes can be mainly attributed to the combined effects of lower relative humidity and higher temperature in the dry curing environment. These conditions accelerate moisture evaporation from the specimen surface, creating a strong moisture gradient between the exterior and interior of the concrete. Because the inward migration of capillary water cannot keep pace with rapid surface evaporation, volumetric contraction is intensified. Moreover, as hydration proceeds, the internal demand for water in UHPC increases, while the limited external moisture supply under dry curing is insufficient to compensate for this consumption. This imbalance leads to elevated capillary tension and internal negative pore pressure, thereby amplifying both drying and autogenous shrinkage. These results highlight the critical importance of the curing environment in controlling the early-age volumetric stability of UHPC. In practical engineering applications, premature exposure of UHPC to dry and warm conditions should be avoided as much as possible, and adequate early-age curing measures should be implemented to reduce shrinkage-induced cracking and to ensure long-term durability.

## 3. Early-Age Shrinkage Prediction Model

### 3.1. Datasets and Input Parameters

Based on the preliminary experimental results, two datasets were established in this study, corresponding to early drying shrinkage and early autogenous shrinkage, each comprising 160 data samples. Seven variables were selected as input features: binder-to-sand ratio (B/S), water-to-binder ratio (W/B), polypropylene fiber volume fraction (PF), curing environment (CE), water content (W), river sand content (S), and curing age (A). The curing environment was encoded numerically, with standard curing assigned a value of 1 and dry curing a value of 2. The measured drying shrinkage and autogenous shrinkage at each curing age were taken as the respective output target variables for model development. Compared with typical machine learning studies that rely on large datasets, the experimental database established in this study represents a relatively small but systematically controlled dataset derived from a well-defined experimental program, which allows for the reliable investigation of parameter interactions affecting UHPC shrinkage behavior.

To construct the dataset, cubic specimens were prepared for each mixture proportion. Length changes were measured at predetermined curing ages, and each measurement at a specific age was treated as an individual data sample. Consequently, multiple samples originated from the same specimen or from replicate specimens of the same mixture, resulting in inherent grouping structure within the dataset. To prevent data leakage where information from the same mixture appears in both training and test sets, the dataset was split based on mixture proportions rather than individual samples. Specifically, all samples from a given mixture were assigned entirely to either the training set or the test set. The training set comprised samples from 80% of the total samples), while the test set comprised samples from 20% of the total samples. This grouped splitting strategy ensures that the generalization performance of the model is evaluated on entirely unseen mixtures, providing a more realistic and reliable assessment. Considering the differences in magnitude and dimensionality among the input parameters, all input and output variables were preprocessed using min–max normalization, which linearly scales the data into the [0, 1] range. This normalization procedure helps prevent features with larger numerical ranges from dominating the learning process and contributes to improved numerical stability and convergence of the predictive models. Prior to model training, the dataset was examined to ensure data reliability. Since the data were obtained from a controlled experimental program, no missing values were observed and no additional data filtering was required. Outlier inspection was performed by examining the distribution of shrinkage values at different curing ages, and all data points were found to fall within physically reasonable ranges. In addition, a preliminary correlation inspection among input variables was conducted to avoid potential multicollinearity. The selected variables represent independent mixture design parameters and environmental conditions; therefore, they were directly used as model inputs after normalization.

The normalization function is given as follows:

(1) $$x^{\prime }=\frac{x-\min\left(x\right)}{\max\left(x\right)-\min\left(x\right)}$$

This ensures uniform weight scales for all features during training, thereby enhancing both model convergence speed and prediction accuracy.

### 3.2. Introduction to Machine Learning Algorithms

XGBoost is a high-performance ensemble learning algorithm based on Gradient Boosting Trees, widely used in classification, regression, and ranking tasks [14,26]. It iteratively trains multiple weak learners, where each iteration focuses on learning and optimizing the residual of the previous model. By constructing new decision trees through gradient descent, the algorithm progressively improves predictive accuracy [28]. The prediction of XGBoost is implemented via an objective function, as shown in Equation (1).

(2) $$\Gamma \left(\theta \right)=\sum_{i=1}^{n}L\left(yi,\hat{y}i\right)+\sum_{k=1}^{k}\Omega \left(fk\right)$$

In the formula, $\sum_{i=1}^{n}L\left(yi,\hat{y}i\right)$ is the loss function, such as mean squared error (MSE) or (LogLoss). $\sum_{k=1}^{k}\Omega \left(fk\right)$ is the regularization term, commonly including L1 and L2 regularization, which controls the model’s complexity.

Compared with conventional machine learning models such as BPNN, random forest, and SVM, XGBoost offers several distinct advantages that are particularly relevant to the current dataset. These include its gradient boosting framework, which iteratively optimizes residuals using second-order gradient statistics and enables efficient approximation of complex nonlinear relationships between input variables and shrinkage responses. In addition, its built-in regularization mechanism combines L1 and L2 penalties to effectively control model complexity and mitigate overfitting, a critical consideration when working with small-sample experimental datasets. Furthermore, XGBoost exhibits strong robustness under limited data conditions by leveraging tree-based ensemble structures that are less sensitive to feature scaling and outliers. Consequently, XGBoost is especially well-suited to the current dataset, which comprises a relatively small number of systematically designed mixture samples and exhibits strong nonlinear couplings between the input features and shrinkage outputs.

**Figure 8:**
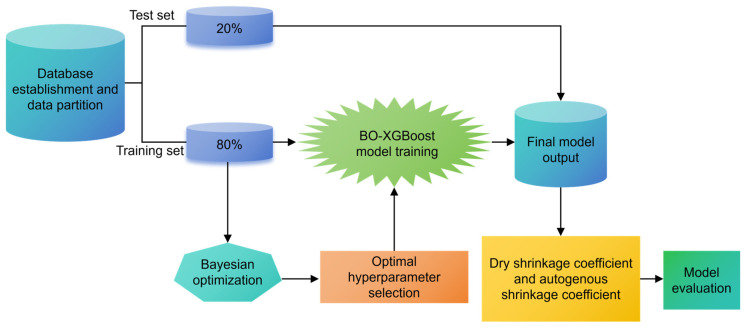
The primary workflow for establishing the model using the BO-XGBoost algorithm.

As a representative gradient-boosted decision tree algorithm, XGBoost involves multiple hyperparameters such as maximum tree depth, number of trees, and learning rate that jointly govern the model’s fitting capacity and generalization performance. Improper selection of these hyperparameters may compromise its advantages, leading to either overfitting due to excessive model complexity or underfitting caused by insufficient learning capacity. Therefore, effective hyperparameter tuning is essential to fully realize the potential of XGBoost for the present modeling task. In practice, manual or grid-based tuning of these parameters is highly time-consuming and often inefficient, particularly when the parameter space is large. Bayesian optimization (BO) provides an effective alternative by constructing a probabilistic surrogate model of the objective function and iteratively updating it to guide the search toward the global optimum with a limited number of evaluations [26,37]. By balancing exploration and exploitation, BO enables efficient identification of near-optimal hyperparameter combinations while significantly reducing computational cost. Accordingly, Bayesian optimization was employed in this study to optimize the hyperparameters of the XGBoost model [35]. The overall workflow of the proposed BO-XGBoost framework, including data input, hyperparameter optimization, model training, and performance evaluation, is schematically illustrated in Figure 8.

All machine learning models were implemented in Python 3.10.12. The XGBoost algorithm was implemented using the XGBoost library. Bayesian optimization was performed with the Scikit-Optimize package 0.9.0. The optimization objective was to minimize the root mean square error (RMSE) of the predictions.

**Figure 9:**
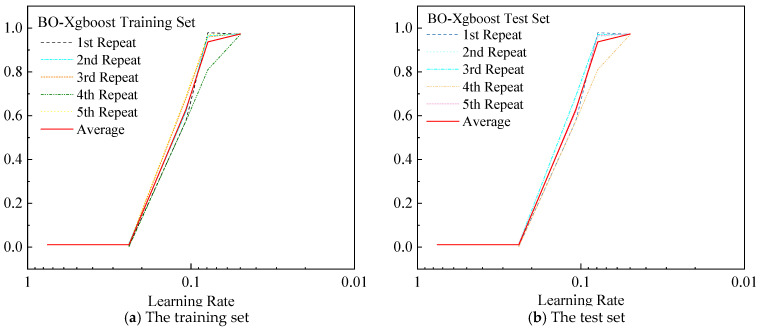
Effect of learning rate on the fitting of the BO-XGBoost model.

In machine learning projects, 5-fold cross-validation balances computational efficiency and estimation stability. Compared with 3-fold cross-validation, it reduces variance and provides a more reliable assessment. Compared with 10-fold cross-validation, it requires fewer training runs and saves resources for large datasets or complex models. Thus, 5-fold cross-validation is an ideal compromise for grid search or Bayesian tuning. In the BO-XGBoost prediction model, Bayesian optimization used 5-fold cross-validation to evaluate each hyperparameter combination. As shown in Figure 9, the model performance varied noticeably with the learning rate, and a value of 0.05 provided the best balance between prediction accuracy and stability across repeated runs. In the five-fold cross-validation, for each candidate learning rate, the prediction error on the validation sets was calculated across the five folds, and the results showed that a learning rate of 0.05 achieved the lowest average error and the smallest fluctuation among folds. The four optimized key hyperparameters were a learning rate of 0.05, a maximum tree depth of 5, 150 estimators, and a subsample ratio of 0.8. A total of 50 iterations were performed to find the combination that minimizes the cross-validation prediction error.

### 3.3. Model Evaluation Indicators

To evaluate the prediction accuracy and generalization performance of each model, the determination coefficient (R^2^), root mean square error (RMSE), and mean absolute error (MAE) were adopted as primary performance metrics, with their calculation formulas as follows:

(3) $$R^{2}=1-\frac{\sum_{i=1}^{n}(y_{i}-{\hat{y}}_{i}{)}^{2}}{\sum_{i=1}^{n}(y_{i}-\bar{y}{)}^{2}}$$

(4) $$RMSE=\sqrt{\frac{1}{n}\sum_{i=1}^{n}(y_{i}-{\hat{y}}_{i}{)}^{2}}$$

(5) $$MAE=\frac{1}{n}\sum_{i=1}^{n}|y_{i}-{\hat{y}}_{i}|$$

Here, $y_{i}$ denotes the measured value, ${\hat{y}}_{i}$ represents the corresponding predicted value, $\bar{y}$ is the mean of the measured values, and N is the total number of samples. The coefficient of determination ($R^{2}$) is used to evaluate the goodness of fit between the predicted and measured data, with values closer to 1 indicating stronger predictive capability. The root mean square error (RMSE) and mean absolute error (MAE) quantify the deviation between predicted and measured values; smaller RMSE and MAE values correspond to lower prediction errors and higher model accuracy.

### 3.4. Comparison of Machine Learning Model Predictions

#### 3.4.1. Early Drying Shrinkage Model of UHPC

**Figure 10:**
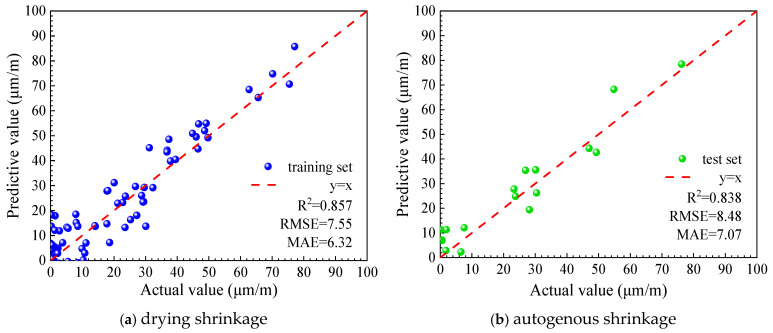
Prediction results of the XGBoost.

**Figure 11:**
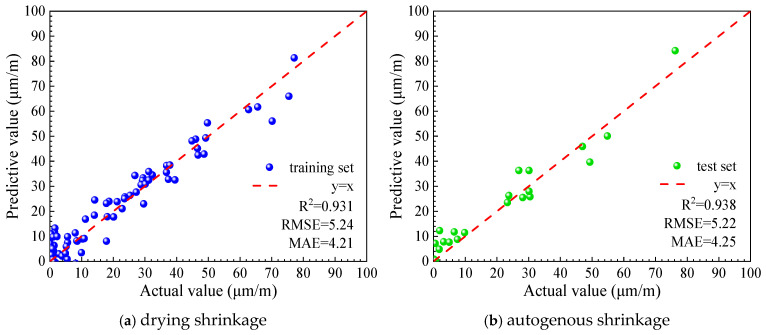
Prediction results of the BO-XGBoost.

Two machine learning models were developed using the UHPC early drying shrinkage dataset to predict early-age drying shrinkage, and both exhibited satisfactory predictive capability. The prediction results for the training and testing datasets are illustrated in Figure 10 and Figure 11, respectively, while the corresponding quantitative performance indicators for each model are summarized in Table 2.

As illustrated in Figure 10 and Figure 11 and summarized in Table 2, both predictive models exhibit good agreement between the predicted and measured values. The coefficients of determination ($R^{2}$) for the XGBoost and Bayesian-optimized XGBoost models are 0.838 and 0.931, respectively, indicating that the Bayesian-optimized model achieves notably higher prediction accuracy for early drying shrinkage of UHPC. In addition, the close consistency of model performance between the training and testing datasets suggests that neither underfitting nor overfitting is evident, confirming the robustness and reliability of the developed models.

#### 3.4.2. Early Autogenous Shrinkage Model of UHPC

**Figure 12:**
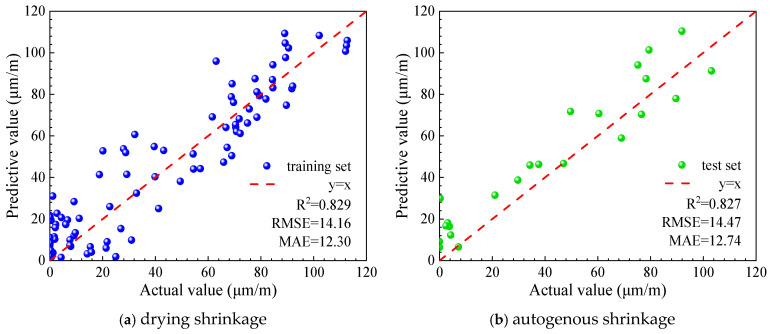
Prediction results of the XGBoost.

**Figure 13:**
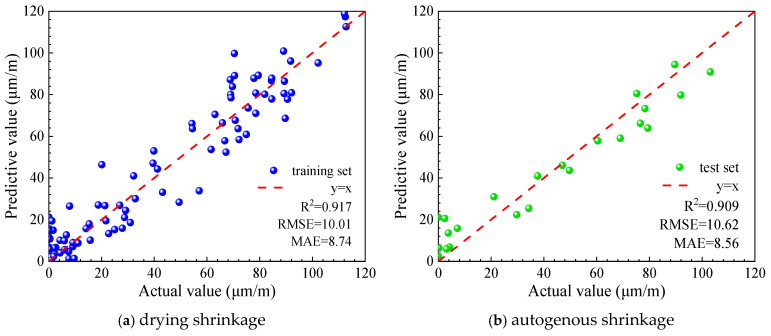
Prediction results of the BO-XGBoost.

Two machine learning models were developed using the UHPC early-age shrinkage dataset to predict the early-age shrinkage behavior of ultra-high-performance concrete. Both models exhibited strong predictive capability. The prediction results for the training and testing datasets are shown in Figure 12 and Figure 13, respectively, and the corresponding performance indicators of the two models are summarized in Table 3.

As illustrated in Figure 12 and Figure 13 and summarized in Table 3, both predictive models show good agreement between predicted and measured values. The coefficients of determination ($R^{2}$) for the XGBoost and Bayesian-optimized XGBoost models are 0.827 and 0.909, respectively, demonstrating that the Bayesian-optimized model achieves higher prediction accuracy for early autogenous shrinkage of ultra-high-performance concrete (UHPC). Moreover, the comparable performance observed for the training and testing datasets indicates that the models exhibit neither underfitting nor overfitting, confirming their stability and generalization capability.

### 3.5. Results and Discussion

#### 3.5.1. Results

In contrast to empirical shrinkage models such as ACI 209 or CEB-FIP, which rely on predefined functional forms and may not fully capture the coupled effects among mixture parameters and curing conditions in UHPC, the proposed BO-XGBoost model provides a data-driven alternative that can better handle nonlinear interactions and small-sample conditions.

To verify the effectiveness of the XGBoost algorithm in predicting the early-age shrinkage behavior of UHPC, several conventional machine learning models were adopted for comparative analysis, including backpropagation neural networks (BPNNs), Random Forest (RF), and Support Vector Machine (SVM) with a radial basis function kernel. These models were trained using the same dataset and evaluation criteria to ensure a fair comparison.

**Figure 14:**
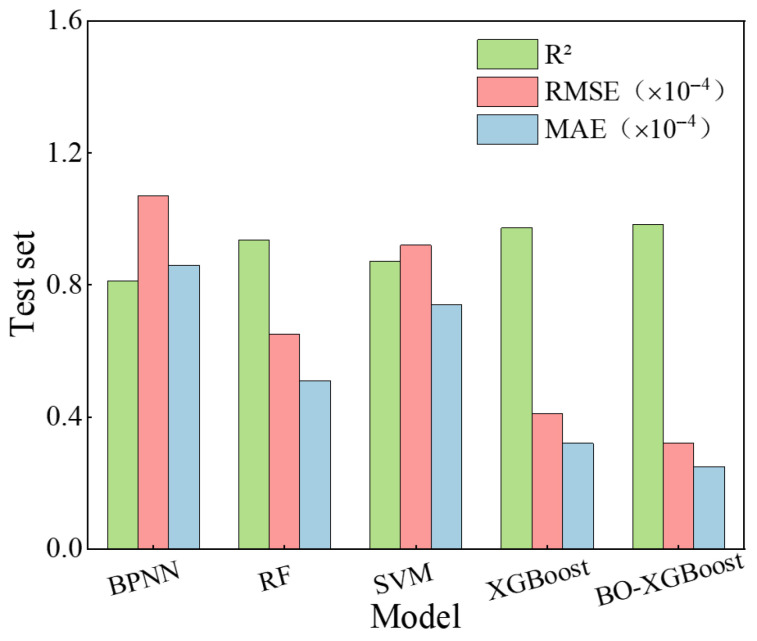
Comparison of prediction performance for early drying shrinkage of UHPC by different models (Test set).

**Figure 15:**
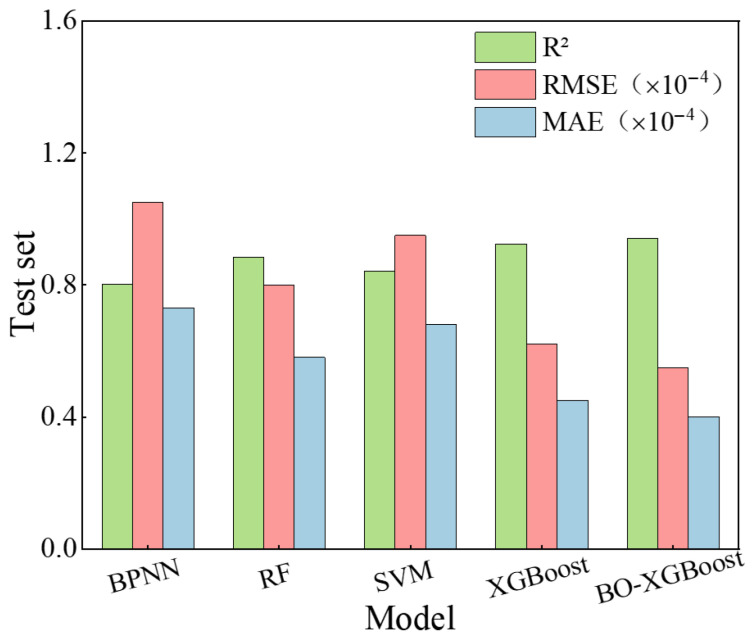
Comparison of prediction performance for early autogenous shrinkage of UHPC by different models (Test set).

Figure 14 and Figure 15 present the prediction performance metrics of the different machine learning models on the UHPC early drying shrinkage and early autogenous shrinkage test sets, respectively. The comparative results provide a quantitative assessment of the relative accuracy and robustness of each algorithm in modeling early-age shrinkage behavior.

As shown in Figure 14 and Figure 15, XGBoost-based models exhibit clear advantages in predicting early drying shrinkage and autogenous shrinkage of UHPC. Both the standard XGBoost and Bayesian-optimized XGBoost models achieve the highest $R^{2}$ values and the lowest RMSE and MAE among all benchmark algorithms, demonstrating superior accuracy in handling small-sample, multiparameter, and strongly nonlinear regression problems. The Bayesian-optimized XGBoost model further improves prediction performance, indicating that Bayesian optimization can efficiently identify near-optimal hyperparameter combinations and enhance model robustness and generalization compared with manual or grid-based tuning methods [24,26].

In contrast, the BPNN model shows the poorest performance, likely due to its sensitivity to local optima and limited generalization ability under small-sample conditions [37]. The random forest model provides moderate accuracy, outperforming BPNN and SVM but remaining inferior to XGBoost, while the SVM model exhibits average predictive capability for the present dataset.

**Figure 16:**
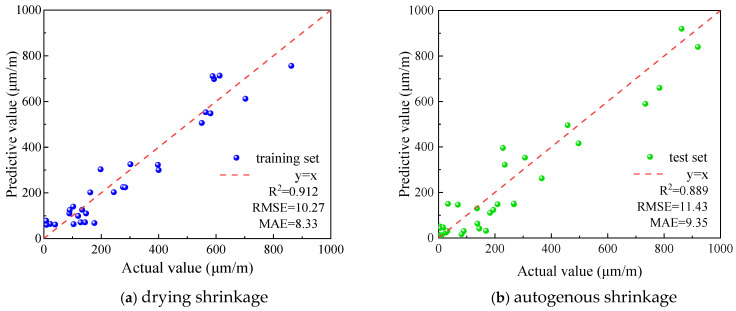
Prediction results of the BO-XGBoost.

To further verify the generalization ability and physical consistency of the proposed BO-XGBoost model under different experimental conditions, this study conducted external validation using independent shrinkage datasets from published literature in an existing database. The external data were directly used as test inputs for the pre-trained model without retraining or fine-tuning, thereby rigorously evaluating the model’s generalization performance. The external validation results are shown in Figure 16. For drying shrinkage, the model achieved an R^2^ of 0.912 and an RMSE of 10.27 on the external data. For autogenous shrinkage, the R^2^ was 0.889 and the RMSE was 11.43. These results are generally comparable to those obtained on the internal test set, indicating that the model maintains high prediction stability on external data.

These findings demonstrate that the model does not overfit the training data and can effectively capture the nonlinear relationships among mix proportion parameters, curing conditions, and shrinkage responses. The successful external validation further confirms the practical value of the BO-XGBoost model in engineering applications, providing reliable support for early-stage mix proportion design and shrinkage risk assessment.

Feature importance analysis reveals that curing age (A) and curing environment (CE) are the dominant factors governing early drying shrinkage, whereas curing age (A) and water-to-binder ratio (W/B) primarily control early autogenous shrinkage. The binder-to-sand ratio (B/S) also plays a secondary role in both cases, while the influence of polypropylene fiber content (PF) is comparatively weaker. These results are consistent with the physical mechanisms of moisture loss, self-desiccation, and pore structure evolution in UHPC, confirming the interpretability and reliability of the proposed modeling approach.

#### 3.5.2. Discussion

To gain deeper insight into the predictive behavior of the Bayesian-optimized XGBoost (BO-XGBoost) model and to quantitatively assess the effects of mix design and curing parameters on early-age shrinkage of UHPC, the SHAP (Shapley Additive Explanations) framework was employed. By computing Shapley values, SHAP provides a consistent and theoretically grounded measure of the contribution of each input variable to the model output, thereby converting the model from a purely data-driven “black box” into an interpretable tool with clear physical relevance. This approach enables interpretation at both the global level, by revealing overall feature importance, and the local level, by explaining individual predictions in terms of underlying variables.

**Figure 17:**
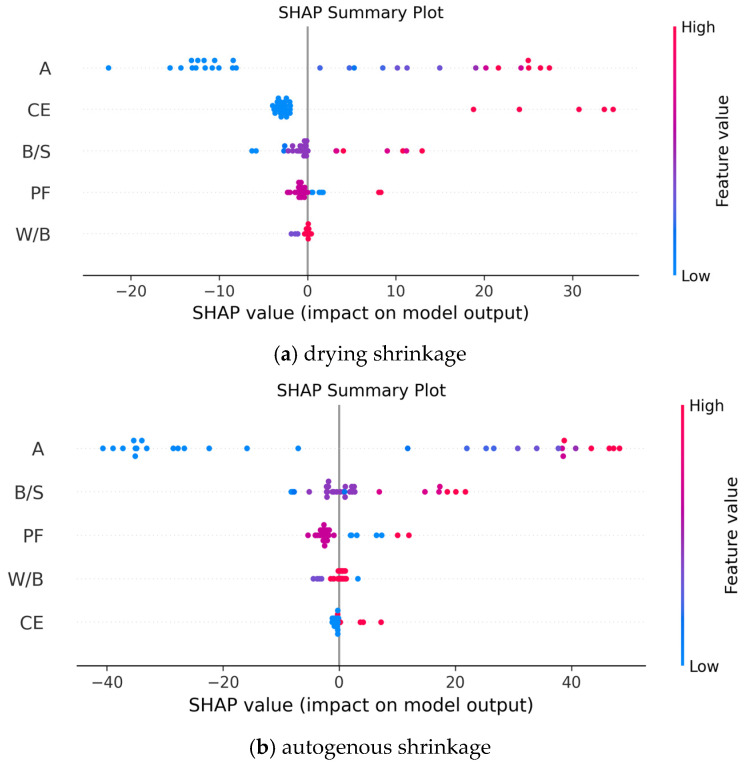
The SHAP summary plots for the shrinkage of UHPC.

**Figure 18:**
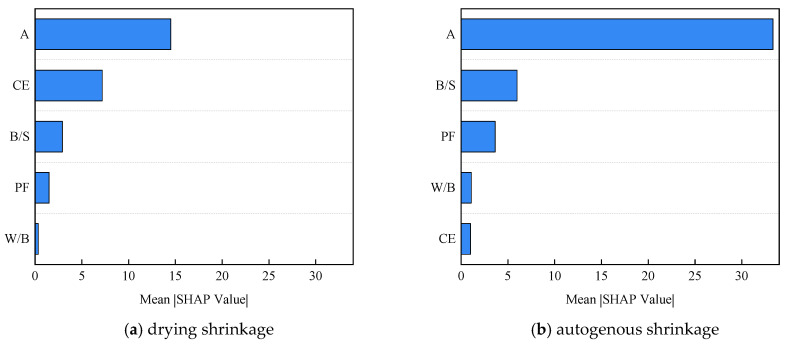
SHAP values for early-age shrinkage of UHPC.

Based on the trained BO-XGBoost model, SHAP values were calculated and visualized separately for early drying shrinkage and autogenous shrinkage. As illustrated in the SHAP summary plots in Figure 17 and Figure 18, feature importance was determined by ranking the input variables according to the mean absolute SHAP values over all samples, providing a quantitative measure of their relative influence on early-age shrinkage behavior.

Based on the SHAP framework, this study quantitatively analyzed the key factors governing the prediction of early-age shrinkage of UHPC and clarified their underlying mechanisms. For early drying shrinkage, feature importance follows the order curing age, curing environment, binder-to-sand ratio, polypropylene fiber content, and water-to-binder ratio. This ranking indicates that drying shrinkage is predominantly controlled by time-dependent processes and external environmental conditions. Notably, the curing environment exerts a stronger influence than any individual mix-design parameter, highlighting that moisture evaporation under dry conditions is the primary driving mechanism of drying shrinkage, outweighing the effect of compositional adjustments. This observation is consistent with the physical mechanism of drying shrinkage in cement-based materials. Under dry curing conditions, moisture evaporation from the pore structure leads to the development of capillary tension within the cement matrix, which results in volumetric contraction of the material. Therefore, curing environment and curing age play dominant roles in controlling drying shrinkage behavior.

In contrast, early autogenous shrinkage is mainly influenced by curing age, followed by binder-to-sand ratio, polypropylene fiber content, and water-to-binder ratio, while the curing environment plays a relatively minor role. This pattern reflects the intrinsic nature of autogenous shrinkage, which is governed primarily by internal hydration reactions and self-desiccation processes rather than external humidity conditions. During hydration, the consumption of internal water reduces the internal relative humidity of UHPC. This process generates capillary pressure in the pore structure and leads to autogenous shrinkage deformation. Consequently, parameters such as curing age and water-to-binder ratio become the dominant factors influencing autogenous shrinkage development. SHAP dependence analysis further reveals distinct response characteristics of individual variables. Curing age exhibits a consistently positive contribution in both models, consistent with the cumulative development of shrinkage over time. In the drying shrinkage model, a shift from standard to dry curing conditions results in a pronounced positive increase in SHAP values, quantitatively confirming the accelerating effect of dry environments. By contrast, the influence of curing environment on autogenous shrinkage remains limited. Increasing the binder-to-sand ratio leads to higher SHAP values in both models, with a more pronounced effect on autogenous shrinkage, indicating that higher paste content intensifies internal volumetric contraction. The effect of polypropylene fiber content is distinctly nonlinear, with the strongest shrinkage mitigation observed at approximately 0.10%, confirming the existence of an optimal fiber dosage. The water-to-binder ratio shows a negative correlation with shrinkage contribution, particularly for autogenous shrinkage, where SHAP values increase sharply when the ratio falls below 0.16, reflecting intensified self-desiccation at low water availability. A lower water-to-binder ratio generally results in a finer pore structure and higher capillary pressure during hydration, which intensifies shrinkage deformation in UHPC. Interaction analysis provides additional insight into coupled effects between variables. For drying shrinkage, the combination of dry curing conditions and a high binder-to-sand ratio produces a synergistic increase in predicted shrinkage, representing a high-risk scenario. For autogenous shrinkage, the interaction between a low water-to-binder ratio and a high binder-to-sand ratio significantly amplifies shrinkage, indicating the compounded influence of internal material parameters.

These results indicate that the machine learning model successfully captures the underlying physical processes governing UHPC shrinkage behavior. Overall, SHAP analysis substantially enhances the interpretability and credibility of the BO-XGBoost model by quantitatively linking data-driven predictions with established material mechanisms. The results clearly distinguish the governing principles of the two shrinkage types: drying shrinkage is dominated by external moisture transport, whereas autogenous shrinkage is controlled by internal hydration and self-desiccation. Moreover, SHAP-based nonlinear and interaction analyses provide practical guidance for mix design and curing optimization, enabling informed trade-offs among shrinkage control, strength development, and durability. From the perspective of explainable artificial intelligence, this approach offers a robust quantitative framework for understanding and controlling early-age shrinkage in UHPC.

The proposed BO-XGBoost model provides a reliable, interpretable tool for optimizing UHPC mix design, enhancing structural durability, and controlling early-age cracking. Quantifying the effects of key parameters such as curing condition, water-to-binder ratio, and binder-to-sand ratio on shrinkage helps prioritize the most critical design variables. For drying shrinkage, improving curing conditions should take priority. For autogenous shrinkage, reducing the water-to-binder ratio or optimizing the binder-to-sand ratio is more effective. SHAP-based interaction analysis further identifies high-risk coupled effects, such as dry curing combined with a high binder-to-sand ratio, offering guidance to avoid unfavorable design combinations. Overall, the framework achieves high prediction accuracy while bridging data-driven modeling with performance-oriented design, demonstrating strong practical engineering potential.

## 4. Conclusions

Based on the systematic experimental investigation and machine learning modeling conducted in this study, the following conclusions can be drawn:

(1) Both early drying shrinkage and autogenous shrinkage of UHPC increase significantly with an increasing binder-to-sand ratio and decreasing water-to-binder ratio. Polypropylene fiber shows the most effective shrinkage mitigation at a volume fraction of approximately 0.10%, whereas lower or higher dosages lead to reduced effectiveness. A dry curing environment markedly intensifies drying shrinkage, with its magnitude being about 2.5 times that under standard curing, highlighting the critical role of early-age humidity control.
(2) This study successfully introduces the XGBoost algorithm, particularly the Bayesian-optimized BO-XGBoost model, for high-accuracy prediction of early-age shrinkage in UHPC. Compared with conventional models such as BPNN, RF, and SVM, the BO-XGBoost model exhibits superior prediction accuracy, robustness, and generalization ability, with these advantages being especially pronounced under small-sample conditions. On the test dataset, the model achieved R^2^ values of 0.938 and 0.909 for early drying shrinkage and autogenous shrinkage, respectively, together with the lowest RMSE and MAE values.
(3) Based on the feature importance analysis of the BO-XGBoost model combined with the SHAP interpretation framework, the contribution of each input variable was quantitatively evaluated. For drying shrinkage, the curing environment is identified as the most influential factor, followed by curing age and water-to-binder ratio. For autogenous shrinkage, curing age plays a dominant role, followed by water-to-binder ratio and binder-to-sand ratio. The SHAP analysis further reveals pronounced nonlinear effects, including the existence of an optimal polypropylene fiber dosage range, as well as interaction effects such as the coupling between low water-to-binder ratio and extended curing age. These findings provide data-driven support for established material mechanisms and offer clear guidance for mix proportion optimization.
(4) The results demonstrate the strong potential and practical applicability of the Bayesian-optimized XGBoost algorithm in predicting UHPC performance. The proposed model offers a reliable tool for precision mix design and intelligent control of UHPC, contributing to the mitigation of early-age cracking risk.

Future studies may expand the applicability of the proposed framework by incorporating a broader range of supplementary cementitious materials, fiber types, curing environments, and long-term shrinkage data, thereby further enhancing its predictive capability for durability-related performance.

## Figures and Tables

**Table 1:** Test Mix Proportion and Grouping.

Sample	Binder-Sand Ratio	Water-Binder Ratio	Mass Content of Water Reducer/%	Steel Fiber Volume Content/%	Polypropylene Fiber Volume Content/%	Curing Condition
S1	0.8	0.17	0.8	2	0.10	Standard curing
S2	0.9	0.17	0.8	2	0.10	Standard curing
S3	1.0	0.17	0.8	2	0.10	Standard curing
S4	1.1	0.17	0.8	2	0.10	Standard curing
S5	1.2	0.17	0.8	2	0.10	Standard curing
S6	1.0	0.15	0.8	2	0.10	Standard curing
S7	1.0	0.16	0.8	2	0.10	Standard curing
S8	1.0	0.17	0.8	2	0.05	Standard curing
S9	1.0	0.17	0.8	2	0.10	Dry curing
S10	1.0	0.17	0.8	2	0.15	Standard curing

**Table 2:** Predictive performance of XGBoost and BO-XGBoost models for early drying shrinkage.

Model Types	XGBoost		BO-XGBoost	
	Training Set	Test Set	Training Set	Test Set
RMSE	7.55	8.48	5.22	5.24
MAE	6.32	7.07	4.25	4.21
R^2^	0.857	0.838	0.938	0.931

**Table 3:** Predictive performance of XGBoost and BO-XGBoost models for early autogenous shrinkage.

Model Types	XGBoost		BO-XGBoost	
	Training Set	Test Set	Training Set	Test Set
RMSE	14.16	14.47	10.01	10.62
MAE	12.30	12.74	8.74	8.56
R^2^	0.829	0.827	0.917	0.909

## Data Availability

The original contributions presented in this study are included in the article. Further inquiries can be directed to the corresponding authors.
